# Analgesic effects of combined transversus abdominis plane block and intramuscular electrical stimulation in patients undergoing cytoreductive surgery followed by hyperthermic intraperitoneal chemotherapy: a randomized controlled trial

**DOI:** 10.1097/JS9.0000000000000383

**Published:** 2023-04-03

**Authors:** Hyun-Chang Kim, Jinyoung Park, Jinyoung Oh, Minjae Kim, Eun Jung Park, Seung Hyuk Baik, Young Song

**Affiliations:** a Department of Anesthesiology and Pain Medicine, Anesthesia and Pain Research Institute; b Department of Rehabilitation, Gangnam Severance Hospital; c Department of Anesthesiology and Pain Medicine; d Division of Colon and Rectal Surgery, Department of Surgery, Gangnam Severance Hospital, Yonsei University College of Medicine, Seoul; e Department of Anesthesiology and Pain Medicine, Kyungpook National University Chilgok Hospital, School of Medicine, Kyungpook National University, Daegu, Republic of Korea

**Keywords:** analgesia, cytoreductive surgery, hyperthermic intraperitoneal chemotherapy, intramuscular electrical stimulation, recovery, transabdominal plane block

## Abstract

To evaluate the analgesic efficacy of a four-quadrant transversus abdominis plane (4QTAP) block and a combination of 4QTAP block with needle electrical twitch and intramuscular electrical stimulation (NETOIMS) in patients undergoing cytoreductive surgery (CRS) followed by hyperthermic intraperitoneal chemotherapy (HIPEC).

**Materials and Methods:**

Eighty-one patients who underwent CRS followed by HIPEC were included in this study. Patients were randomly assigned to one of three groups: group 1 (intravenous patient-controlled analgesia, control group), group 2 (preoperative 4QTAP block), and group 3 (preoperative 4QTAP block and postoperative NETOIMS). The primary study endpoint was the pain score assessed by the visual analog scale (VAS: 0, no pain; 10, worst imaginable pain) on postoperative day (POD) 1.

**Results:**

The VAS pain score on POD 1 was significantly lower in group 2 than in group 1 (6.0±1.7 and 7.6±1.9, *P*=0.004), whereas that in group 3 was significantly lower than that in groups 1 and 2 (*P*<0.001 and *P*=0.004, respectively). Opioid consumption and nausea and vomiting incidence during POD 7 were significantly lower in group 3 than in groups 1 and 2. Gait speed and peak cough flow on POD 4 and 7, as well as the quality of recovery (QoR)-40 score on POD 4, were significantly higher in group 3 than in groups 1 and 2.

**Conclusions:**

The combination of a 4QTAP block with NETOIMS provided more effective analgesia than a 4QTAP block alone after CRS, followed by HIPEC, and enhanced functional restoration and quality of recovery.

## Introduction

Highlights

- Cytoreductive surgery followed by hyperthermic intraperitoneal chemotherapy causes severe pain.
- Adequate pain management may enhance recovery after surgery.
- Combined four-quadrant transversus abdominis plane block and needle electrical twitch improved postoperative pain scores and reduced opioid requirements.
- Combined four-quadrant transversus abdominis plane block and needle electrical twitch improved postoperative functionality and quality of recovery.

Cytoreductive surgery (CRS) followed by hyperthermic intraperitoneal chemotherapy (HIPEC) is an important treatment option for improving the survival of select patients with peritoneal metastasis^1^–^3^. Most patients undergoing CRS, followed by HIPEC, experience extreme postoperative pain^4^. Adequate pain control is being increasingly emphasized, and the recently developed enhanced recovery after surgery (ERAS) guideline in CRS followed by HIPEC advises multimodal analgesia consisting of several non-opioid medications, although the evidence and recommendation strength are not strong^4^. Thoracic epidural analgesia (TEA), which is also listed in the ERAS protocol, provides good analgesic effects but has not been implemented universally because of the risk of serious complications and its association with delayed recovery^4^–^8^.

The four-quadrant transversus abdominis plane (4QTAP) block, which is a bilateral subcostal and lateral block, has emerged as an alternative neuraxial block to provide analgesia to the anterior abdominal wall^9^. It has currently been suggested for CRS followed by HIPEC^8^, but evidence of its efficacy is scarce and equivocal. A comparison study between 4QTAP block and TEA revealed a greater requirement of opioids in patients receiving 4QTAP block, although the pain scores and quality of recovery were not inferior to those receiving TEA^9^. A more recent clinical trial reported an overall decrease in opioid requirements in patients receiving a 4QTAP block compared with those who did not receive it, but the pain scores were not reduced^10^. Moreover, the mechanism by which functional recovery or patient-centered outcomes can be improved remains unclear.

Needle electrical twitch obtaining intramuscular stimulation (NETOIMS), which has been used to relieve muscular pain in myofascial pain syndrome or post-stroke spasticity, was recently demonstrated to exert postoperative analgesia in open abdominal^11^ or thoracoscopic lung resection surgery^12^. It targets deep motor endplate zones of skeletal muscles, and its action in relaxing hypercontracted muscles and modulating inflammatory cytokines is thought to be a mechanism of postoperative pain control^12^–^14^. Considering that open CRS followed by HIPEC may result in severe postoperative hypercontraction of the abdominal muscles induced by extensive and prolonged muscle retraction following a long midline incision, we could expect that NETOIMS would play a distinct role in somatic pain control. Given the different action mechanisms with different primary target tissues of treatment (i.e. nerve for 4QTAP and skeletal muscle for NETOIMS), we hypothesized that the addition of NETOIMS to the 4QTAP block could display synergistic effects.

This prospective, randomized, controlled trial aimed to compare postoperative pain intensity and opioid requirements in three groups: no pain block, 4QTAP block, and a combination of 4QTAP block and NETOIMS and to evaluate the effects of these interventions on functional restoration and patient-reported quality of recovery.

## Methods

The work has been reported in line with Consolidated Standards of Reporting Trials (CONSORT) Guidelines. This single-center randomized controlled study was approved by the Institutional Review Board of Yonsei University Health System Gangnam Severance Hospital (IRB 3-2021-0032) and registered at ClinicalTrials.gov (NCT04981639) on 29 July 2021. This center performs more than 70 surgical cases per day. This study was performed in accordance with the tenets of the Declaration of Helsinki. Written informed consent was obtained prior to patient enrollment.

Patients with American Society of Anesthesiologists physical status I–III, aged at least 19 years, and scheduled for CRS followed by HIPEC, were enrolled from August 2021 to May 2022. Patients with a history of abdominal surgery, inability to walk independently owing to musculoskeletal problems, allergy to local anesthetics, history of chronic pain, pacemakers, inability to be extubated, history of drug abuse, pregnancy, and inability to communicate and answer the questionnaire were excluded from this investigation. Two surgeons performing more than 50 CRS followed by HIPEC per year were included.

### Study design

Patients were randomly assigned to groups 1, 2, and 3 in a 1 : 1 : 1 ratio using R Statistical Software (Foundation for Statistical Computing, Vienna, Austria) and the closed-envelope technique preoperatively by a nurse who did not participate in the investigation. Group 1 did not receive any invasive analgesic intervention. Group 2 received a 4QTAP block immediately after anesthesia induction before making the vertical abdominal incision. Group 3 received a 4QTAP block at the same time point as group 2 and received NETOIMS immediately after surgical wound closure, while general anesthesia was maintained. Operators, patients, and physicians who managed the patients postoperatively were blinded to the group allocation and study protocol throughout the study period.

**Figure 1:**
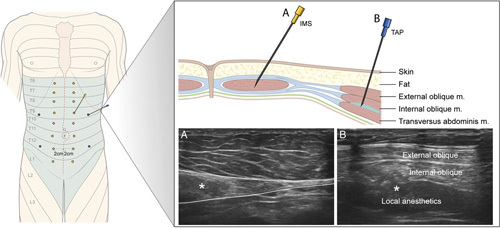
Schematic diagram of needle electrical twitch obtaining intramuscular stimulation and four-quadrant transversus abdominis plane block. (A) Needle electrical twitch obtaining intramuscular stimulation; (B) four-quadrant transversus abdominis plane block. IMS, intramuscular stimulation; TAP, transversus abdominis plane.

This procedure is schematically depicted in Figure 1. The 4QTAP block was performed under ultrasound guidance after anesthesia induction by an experienced anesthesiologist who had performed more than 50 4QTAP blocks, as previously described^15^. Bilateral subcostal blocks were performed by injecting 10 ml of 0.375% ropivacaine with 1:200, 000 epinephrine into the plane between the transversus abdominis and rectus abdominis muscles on both sides. Bilateral lateral blocks were performed by injecting the same amount of the drug into the plane between the transversus abdominis and internal oblique muscles on both sides. NETOIMS was performed under ultrasound guidance after abdominal wound closure by an experienced anesthesiologist who had performed more than 50 NETOIMSs, as previously described^11^. A monopolar needle electrode (Technomed, Amerikalaan, Netherlands) was inserted at 14 stimulation points in the rectus abdominis muscle located ∼2 cm lateral to the incisional wound. At each insertion point, 1 Hz of squared electrical stimulations with 2-mA intensity and 0.2-ms pulse duration were delivered for 10 s through an electrical stimulator, Clavis (Alpine Biomed ApS, Skovlunde, Denmark). We confirmed the correct location using stimulation-induced muscle twitches with ultrasound visualization. We checked the occurrence of any potential complications of the procedures, such as visceral damage, accidental intravascular injection of local anesthetic, and infection.

All anesthetic and surgical management procedures were performed according to our institutional standards, as previously described^16^,^17^. All surgical procedures were performed using an open-method laparotomy, and the duration of HIPEC was 90 min. We used the peritoneal cancer index to assess the extent of peritoneal carcinomatosis^18^. We classified the resection status based on the completeness of the cytoreduction score^18^. The postoperative analgesia and antiemetic protocol provided to all patients included fentanyl (50 μg) and ramosetron (0.3 mg) 20 min before tracheal extubation and intravenous (i.v.) patient-controlled analgesia (PCA) consisting of fentanyl (20 µg/kg) with 0.3 mg of ramosetron (total volume, 100 ml; infusion rate, 2.0 ml/h; bolus, 0.5 ml; lockout time, 15 min), which was started at the end of surgery. If patients complained of pain, with a visual analog scale score (VAS; 0, no pain; 10, worst imaginable pain) exceeding 4, fentanyl (1 μg/kg) at the postoperative analgesia care unit and tramadol (50 mg), pethidine (25 mg), or ibuprofen (400 mg) in the ward were administered as rescue pain medications. Granisetron (1 mg) was administered as antiemetic prophylaxis on postoperative days (POD) 1 and 2.

### Outcome measures

The primary study endpoint was the postoperative VAS pain score on POD 1. The secondary endpoints were VAS pain scores on POD 0, 2, 3, 5, 7, 14, and 28; daily opioid consumption on POD 1–7; the presence of nausea and vomiting on POD 7; peak cough flow (PCF) and gait speed on POD 4, 7, and 14; and quality of recovery (QoR)-40 score on POD 4 and 7. Daily opioid consumption was defined as the sum of the doses administered via i.v. PCA and rescue medication, which was converted to morphine milligram equivalents (MME).

To assess the degree of complications related to pulmonary function, the PCF was consecutively measured. The patients were instructed to cough to the maximum capacity in the 90° sitting position, and a nurse checked the maximum value among three attempts using a peak flow meter (Mini-Wright Standard PFM, Clement Clarke International). Between each measurement, the patient was allowed to rest until cough-induced pain subsided.

The gait speed was measured to determine the degree of functional recovery. The patients walked straight along a 15-m track; the time taken to finish walking was recorded, and the distance-to-time ratio was calculated. The maximum gait speed was recorded in three attempts. To prevent the rater from encouraging each patient to different degrees, the rater stood at the endpoint of the track rather than walking alongside the patient. Both parameters are presented as a percentage relative to the preoperative baseline value measured the day before surgery.

The QoR-40 questionnaire consists of 40 questions that evaluate five fields of patient recovery, including physical comfort, pain, physical independence, psychological support, and emotional status^19^. A higher score represents a better QoR.

Demographic intraoperative data, including the amount of remifentanil administered during the operation and mean arterial pressure (MAP), heart rate immediately before and after surgical skin incision, and postoperative data, were also recorded.

### Statistical analysis

Our pilot study in 20 patients who received CRS followed by HIPEC showed a VAS pain score of 7.0±2.3 on POD 1. We assumed that the 4QTAP block or combined 4QTAP block with NETOIMS would reduce the score by 70%, which was regarded as clinically significant. Nineteen patients per group were required to attain 5% alpha and 80% power in a two-sided comparison. Considering a possible dropout rate of 20% and a compliance rate of 90%, 81 patients were enrolled.

Continuous variables are presented as mean±standard deviation or median (interquartile range), with comparisons made using a one-way analysis of variance or the Kruskal–Wallis test. Categorical variables were presented as the number of patients (proportion) with comparisons using the *χ* ^2^ test or Fisher’s exact test, as appropriate. All statistical analyses were performed using Statistical Package for the Social Sciences version 25 (IBM Corp., Armonk, New York, USA). Statistical significance was defined as a two-sided *P* value of less than 0.05.

## Results

**Figure 2:**
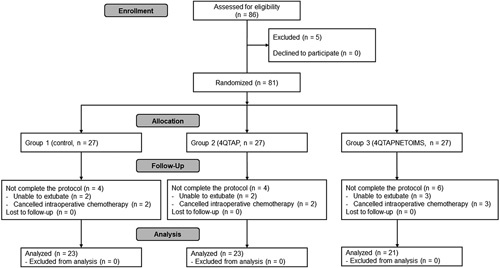
Consolidated Standards of Reporting Trials (CONSORT) diagram. 4QTAP, four-quadrant transversus abdominis plane block; 4QTAPNETOIMS, four-quadrant transversus abdominis plane block combined with needle electrical twitch obtaining intramuscular electrical stimulation

This study screened 86 patients from August 2021 to May 2022, of whom five patients were excluded. Two patients each in groups 1 and 2 and three patients in group 3 were unable to be extubated at the end of anesthesia, and two patients each in groups 1 and 2 and three patients in group 3 did not receive chemotherapy, leaving 67 patients for the final analysis (Figure 2). No complications were attributable to the 4QTAP block or NETOIMS.

Demographic and intraoperative data were not different between the groups, except for intraoperative remifentanil consumption and MAP immediately after skin incision, which were lower in groups 2 and 3 than in group 1 (both *P*<0.001; Table 1). Important harms or unintended effects were not found.

**Table 1:** Baseline patient characteristics.

	Group 1 (*n*=23)	Group 2 (*n*=23)	Group 3 (*n*=21)	*P*
Sex				0.568
Male	11 (48%)	14 (61%)	13 (62%)	
Female	12 (52%)	9 (39%)	8 (38%)	
Age (year)	57±11	55±11	57±15	0.785
Height (cm)	163±10	166±11	164±9	0.622
Weight (kg)	60±12	65±10	64±12	0.450
Body mass index (kg/m^2^)	22.6±3.2	23.4±3.2	23.7±3.9	0.532
Primary cancer				0.162
Pseudomyxoma peritonei	5 (22%)	6 (26%)	1 (5%)	
Appendiceal cancer	4 (17%)	3 (13%)	5 (24%)	
Cecal cancer	1 (4%)	1 (4%)	2 (10%)	
Ascending colon cancer	3 (13%)	2 (9%)	5 (24%)	
Transverse colon cancer	0 (0%)	1 (4%)	0 (0%)	
Descending colon cancer	0 (0%)	0 (0%)	3 (14%)	
Sigmoid colon cancer	8 (35%)	9 (39%)	2 (10%)	
Rectosigmoid colon cancer	1 (4%)	0 (0%)	1 (5%)	
Rectal cancer	1 (4%)	0 (0%)	2 (10%)	
Urachal cancer	0 (0%)	1 (4%)	0 (0%)	
ASA physical status (I/II/III)				0.915
I	11 (48%)	11 (48%)	11 (52%)	
II	10 (44%)	9 (39%)	9 (43%)	
III	2 (9%)	3 (13%)	1 (5%)	
Baseline gait speed (m/s)	1.3±0.4	1.3±0.3	1.2±0.3	0.231
Baseline peak cough flow (l/min)	310±116	372±104	352±114	0.161
Comorbidities				
Hypertension	8 (35%)	7 (30%)	5 (24%)	0.727
Diabetes	3 (13%)	2 (9%)	2 (9%)	0.878
Coronary artery disease	1 (4%)	0 (0%)	0 (0%)	0.379
Arrhythmia	1 (4%)	1 (4%)	0 (0%)	0.625
Kidney disease	0 (0%)	0 (0%)	1 (5%)	0.329
Liver disease	1 (4%)	1 (4%)	0 (0%)	0.625
Thyroid disease	5 (22%)	2 (9%)	2 (9%)	0.353
Operation time (min)	330 (185)	350 (238)	380 (188)	0.820
Anesthesia time (min)	435 (210)	410 (275)	480 (203)	0.698
Cytoreductive surgery time (min)	250 (180)	270 (208)	300 (180)	0.795
Peritoneal cancer index	7 (24)	9 (22)	24 (15)	0.456
Completeness of cytoreduction	0 (1)	0 (1)	3 (2)	0.457
Intraoperative dose of remifentanil (µg/kg/h)	5.2±2.7	3.1±0.8*	3.1±1.1*	<0.001
Intraoperative crystalloid intake (ml/kg/h)	12 (7)	10 (3)	11 (2)	0.120
The amount of intraoperative bleeding (ml)	475 (448)	800 (1175)	850 (1553)	0.714
Length of incision (cm)	30±5	31±4	28±6	0.184
Mean arterial pressure				
1 min before skin incision (mmHg)	74±6	70±8	73±7	0.198
1 min after skin incision (mmHg)	85±14	73±10*	74±4*	<0.001
Heart rate				
1 min before skin incision (beats/min)	65±10	66±8	65±11	0.920
1 min after skin incision (beats/min)	72±11	69±10	70±15	0.661

Values are presented as number of patients (%), mean±standard deviation, or median (interquartile range).

* *P*<0.05, compared to group 1.

ASA, American Society of Anesthesiologists.

### Postoperative pain and outcomes related to analgesics

**Figure 3:**
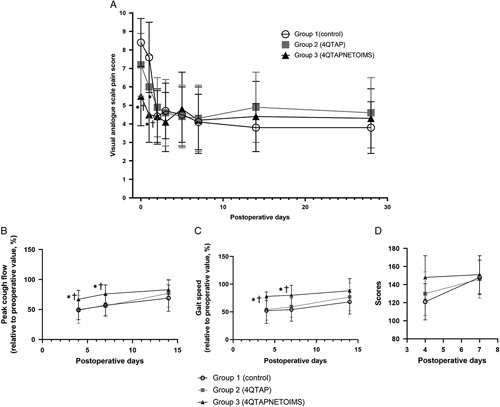
Pain scores, functional outcomes, and quality of life. (A) Pain scores, (B) gait speed, (C) peak cough flow, and (D) quality of recovery-40 scores. 4QTAP, four-quadrant transversus abdominis plane block; 4QTAPNETOIMS, four-quadrant transversus abdominis plane block combined with needle electrical twitch obtaining intramuscular electrical stimulation.

Intergroup differences in postoperative pain are shown in Figure 3A. The VAS pain score was significantly different among the groups on POD 0 and 1 (both *P*<0.001). The score on POD 0 was significantly lower in group 3 (5.5±1.6) than in groups 1 (8.4±1.3, *P*<0.001) and 2 (7.2±1.7, *P*=0.001). The score on POD 1 was significantly lower in group 3 (4.5±1.5) than in groups 1 (7.6±1.9, *P*<0.001) and 2 (6.0±1.7, *P*=0.004), and the difference between groups 2 and 1 was also significant (*P*=0.004).

The amounts of opioids and ibuprofen consumed, the incidence of nausea and vomiting, and functional outcomes are listed in Table 2. Opioid consumption converted to MME was significantly different among the groups on POD 1 and 2 (*P*<0.001 and *P*=0.016, respectively). On POD 1, group 3 required a lower dose (22±10 mg) than group 1 (42±10 mg, *P*<0.001) and group 2 (30±13 mg, *P*=0.025). The difference in MME between the control and group 2 groups was also significant (*P*=0.001). On POD 2, group 3 required a lower dose than group 1 (20±9 mg vs. 29±10 mg, *P*=0.004). Cumulatively, the amount of opioid consumption during the postoperative 7 days was significantly lower in group 3 than in groups 1 (86±34 mg vs. 136±46 mg, *P*<0.001) and 2 (86±34 mg vs. 112±50 mg, *P*=0.046). The total consumption of ibuprofen during the postoperative 7 days was comparable among the groups. The incidence of nausea in group 2 (52%) and group 3 (29%) was significantly lower than in group 1 (91%, *P*=0.007 and *P*<0.001, respectively). The incidence of vomiting in group 2 (9%) and group 3 (14%) was significantly lower than that in group 1 (48%, *P*=0.007 and *P*=0.024. respectively).

**Table 2:** Postoperative variables related to analgesic use and functional outcomes.

	Group 1 (*n*=23)	Group 2 (*n*=23)	Group 3 (*n*=21)	*P*
Morphine milligram equivalents (mg)				
Postoperative day 1	42±10	30±13*	22±10* †	<0.001
Postoperative day 2	29±10	22±12	20±9	0.016
Postoperative day 3	24±13	26±16	16±11	0.059
Postoperative day 4	23±18	22±16	19±15	0.742
Postoperative day 5	5 (10)	5 (10)	3 (5)	0.175
Postoperative day 6	5 (10)	3 (5)	0 (5)	0.297
Postoperative day 7	5 (8)	3 (5)	0 (3)	0.083
Total dose	136±46	112±50	86±34* †	0.002
Ibuprofen consumption (mg)	0 (400)	0 (800)	0 (0)	0.216
Number of patients requiring rescue analgesic medications	15 (65%)	12 (52%)	8 (38%)	0.198
Incidence of nausea	21 (91%)	12 (52%)	6 (29%)	<0.001
Incidence of vomiting	11 (48%)	2 (9%)	3 (14%)	0.004
Time to eat soft food (day)	6 (3)	7 (3)	7 (3)	0.351
Time to discharge (day)	12 (5)	14 (7)	14 (7)	0.264
Gait speed (%)				
Postoperative day 4	52±23	54±19	78±8* †	<0.001
Postoperative day 7	54±21	59±19	80±18* †	<0.001
Postoperative day 14	68±22	77±25	88±22	0.024
Peak cough flow (%)				
Postoperative day 4	49±16	50±23	67±15* †	0.004
Postoperative day 7	57±17	56±18	76±15* †	<0.001
Postoperative day 14	69±22	77±23	83±16	0.082

Values are presented as mean±standard deviation, median (interquartile range), or number of patients (%).

* *P*<0.05, compared to group 1.

† *P*<0.05, compared to group 2.

### Functional outcomes and quality of life

Functional recovery and QoR are shown in Table 3 and Figure 3, respectively. Gait speed was significantly higher in group 3 than in group 1 and group 2 on POD 4 (*P*<0.001 and *P*<0.001, respectively) and POD 7 (*P*<0.001 and *P*=0.001, respectively). The PCF was significantly higher in group 3 than in group 1 and group 2 on POD 4 (*P*=0.001 and *P*=0.006, respectively) and POD 7 (*P*<0.001 and *P*<0.001, respectively). The global QoR-40 score was significantly higher in group 3 than in group 1 and group 2 on POD 4 (*P*<0.001 and *P*=0.022, respectively). The scores of the five subcategories were all significantly higher in group 3 than in group 1. Group 3 also showed significantly higher scores for physical comfort (46±6 vs. 41±8, *P*=0.023) and physical independence (14±4 vs. 10±4, *P*=0.001) than group 2. The global and subcategory scores on POD 7 were comparable among the three groups.

**Table 3:** Quality of recovery-40 score on postoperative days 4 and 7.

	Group 1 (*n*=23)		Group 2 (*n*=23)		Group 3 (*n*=21)		*P*	
QoR-40	POD 4	POD 7	POD 4	POD 7	POD 4	POD 7	POD 4	POD 7
Global	121±20	148±19	130±24	146±21	148±24* †	151±21	0.001	0.706
Emotional status	27±6	32±5	30±6	32±7	33±7*	33±7	0.026	0.993
Physical comfort	39±6	45±6	41±8	45±6	46±6* †	47±6	0.002	0.285
Psychological support	21±4	27±5	23±5	26±5	26±6*	28±5	0.011	0.249
Physical independence	10±4	15±4	10±4	14±4	14±4* †	15±4	0.001	0.578
Pain	24±5	29±4	27±5	29±4	29±5*	27±6	0.007	0.430

Values are presented as mean±standard deviation.

QoR, quality of recovery; POD, postoperative day.

* *P*<0.05, compared to group 1.

† *P*<0.05, compared to group 2.

## Discussion

In this prospective randomized controlled study on patients undergoing CRS followed by HIPEC, we observed that combining the NETOIMS postoperatively with preoperative 4QTAP block reduced subjective pain scores and opioid consumption compared with no block and 4QTAP block without NETOIMS. This combined intervention also improved gait speed, effective coughing capacity, and patient-reporting QoR. The 4QTAP block without NETOIMS could reduce the pain score and opioid consumption compared with no block on POD 1, but it did not result in an improvement of functional measures and QoR. The 4QTAP block reduced the incidence of postoperative nausea and vomiting, regardless of its combination with NETOIMS.

The transversus abdominis plane (TAP) block has gained popularity for decades as an effective analgesic technique in various abdominal surgeries^20^. However, several recent clinical trials and meta-analyses have reported equivocal or modest efficiency and sparked renewed discussion about its use according to surgical type and adjunctive pharmacologic or nonpharmacologic interventions^21^–^23^. Regarding CRS followed by HIPEC, several centers have already implemented the TAP block as their institutional ERAS protocol^9^,^24^; however, its efficacy remains unknown. The first clinical trial on the 4QTAP block compared with TEA reported greater opioid requirements in the 4QTAP block group, while there was no ‘no block’ group as a reference^9^. A more recent study evaluating the effects of a 4QTAP block versus no block showed reduced opioid use and similar pain scores between the groups, and the block was performed after surgery, which is far less efficient and not clinically applicable^10^. Therefore, our findings are the first to show the analgesic effect of a preemptive TAP block compared with no block and showed reduced pain scores and opioid requirements, although the effect only lasted for a day. The TAP block targets the anterior branches of the spinal nerves that dominate the sensation of the skin, muscles, and the parietal peritoneum^25^. As the 4QTAP block covers T6-L1^25^, it would theoretically provide excellent somatic analgesia for open CRS followed by HIPEC, which requires a xiphoid-to-pubis incision. The TAP block also reduces central sensitization, an essential mechanism of postoperative acute and chronic pain, by blocking afferent neural inputs^26^. We could assume that the 4QTAP block in our study could have blocked central sensitization to some extent since the increase in blood pressure by surgical incision was significantly less than that with no block. Moreover, opioid-induced hyperalgesia, which is an important concern related to postoperative pain, could have been attenuated by the 4QTAP block because the amount of remifentanil administered during surgery was significantly reduced. Nevertheless, the 4QTAP block seems to be insufficient to provide satisfactory analgesia after CRS followed by HIPEC, considering that it was only modestly effective and for a short time, while most of this surgical cohort suffered from moderate to severe pain that continued over the weeks. Moreover, pain on the day of surgery, which may be one of the most dreadful experiences, was not reduced by the 4QTAP block alone. In addition, the 4QTAP block alone was not helpful for postoperative ambulation or effective cough. The lack of improvement in the global QoR score may reflect insufficient pain control and a lack of functional restoration in this group.

The addition of NETOIMS immediately after surgery to the preemptive 4QTAP block, which is the first attempt to combine the two simple, feasible procedures for major general surgery, resulted in relatively successful analgesic efficacy. Patients’ subjective pain ratings were effectively reduced to a modest degree on the day of surgery, which was maintained the following day. Although the pain score did not decrease thereafter, opioid consumption was meaningfully reduced for up to 3 days, and the same tendency was maintained for up to 7 days, thereby reducing the total consumption for a week, which might be attributable to a significant decrease in the incidence of nausea. NETOIMS produces a local twitch of targeted muscles, leading to stretching of the shortened muscle fibers, which is its mechanism of action to alleviate muscle pain^13^. Intramuscular electrical stimulation in the skeletal muscle has been demonstrated in the past literature to induce neural decompression of adjacent nerves and lead to recovery of the range of motion by relaxation of abnormally hypercontracted muscles^27^,^28^. Therefore, it can be inferred that intramuscularly administered electrons immediately separate the aberrant actin–myosin cross-bridge in the resting state^11^. Previously reported improved local blood flow and prompt washout of inflammatory cytokines may be followed by the relaxation of myofibrils, which relieves the intrafascial pressure in a restricted cross-sectional area^29^,^30^. It is also possible that irritation of the C-fibers, which could be induced by the increased intrafascial pressure and subsequent relatively ischemic conditions, would be resolved by the relaxation of the myofibrils. Because of the inevitably prolonged abdominal wall retraction for CRS followed by HIPEC, as well as a very long incision and suturing wound, this surgical cohort definitely suffers from abnormal hypercontraction of muscles and consequent pain and stiffness; thus, NETOIMS would have played a role in analgesia. In addition, especially during the HIPEC procedure, the abdominal muscles are pulled and fixed tightly over 90 min, which may interrupt blood flow and subsequently cause additional ischemic pain. The twitch-induced exercise effect of NETOIMS can improve macrocirculation and microcirculation that would restore tissue perfusion^31^, which might be another important mechanism of analgesia in the current study. Since opioids usually interrupt the physical performance, including ambulation, less opioid consumption and lower pain scores on the early postoperative days could have enhanced the recovery of gait speed in the 4QTAPNETOIMS group. Cough capacity was also negatively influenced by pain and opioid administration, which could be the reason why it was significantly greater in this group. In addition, relaxation of the rectus abdominis muscles by NETOIMS could have helped recruit power during coughing^11^. The patient-reported QoR on POD 4 was also significantly improved in this group. Reductions in pain intensity, opioid consumption, and nausea, as well as earlier functional restoration, are in line with the findings that all of the subdimensions of QoR-40 questions reflecting physical and emotional well-being were better in this group. Since adequate gait speed, PCF, and QoR-40 score are reliable indicators of early recovery after abdominal surgery and are known to predict postoperative complications and morbidity^32^–^34^, our findings clearly suggest the clinical benefit of this combination of procedures.

Since the good effect and zero risk of complications for NETOIMS have already been described in open pancreaticoduodenectomy and thoracoscopic surgery^11^,^12^, we tested its impact in combination with the 4QTAP block in this trial. CRS, followed by HIPEC, is characterized by severe postoperative nociceptive pain through huge surgical injuries and inflammation and requires the best efforts of clinicians to provide analgesia. An effective ERAS protocol should be preceded by the best multimodal analgesic strategy that can be provided. In addition to attenuating pain perception by the nerve block, it is important to directly manage postoperative abnormal contraction of skeletal muscles. In this regard, our findings suggest that these two procedures, which alleviate somatic pain via different mechanisms, could be performed in combination with this surgical cohort.

This study has some limitations. Our pain management protocol did not include routine administration of non-opioid agents, which can be regarded as countering contemporary trends in perioperative analgesic regimens. Moreover, analgesia provided by i.v. PCA might not have been sufficient in some patients because the PCA regimen was composed of a relatively low dose of fentanyl, with consideration for the safety issue. Therefore, our results need to be confirmed in the condition of pharmacologic multimodal analgesia or a generous amount of opioids for PCA in further studies. In addition, we did not evaluate the baseline QoR-40 scores. Although baseline demography and disease-related parameters, which may dominate the preoperative score, were not different among the groups, we cannot completely exclude possible bias induced by baseline imbalance.

In conclusion, preemptive 4QTAP may provide analgesia for a while after CRS followed by HIPEC, although it will not significantly enhance recovery. In addition, combining 4QTAP and NETOIMS effectively decreased postoperative pain scores and opioid requirements. Moreover, it promoted functional restoration, which was assessed objectively and improved the QoR of the patients. We recommend this multimodal intervention approach for major abdominal surgeries, including CRS, followed by HIPEC.

## Ethical approval

The study protocol was approved by the Institutional Review Board and Hospital Research Ethics Committee of Gangnam Severance Hospital (#3-2021-0032) in July 2021.

## Sources of funding

This study was supported by the Basic Science Research Program through the National Research Foundation of Korea (NRF), funded by the Ministry of Science and ICT (NRF-2022R1A2C1013201).

## Author contribution

H.-C.K., J.P., J.O., M.K., E.J.P., S.H.B., and Y.S.: conceptualization; H.-C.K., E.J.P., and Y.S.: data curation; H.-C.K., E.J.P., and Y.S.: formal analysis; E.J.P.: funding acquisition; H.-C.K., E.J.P., S.H.B., and Y.S.: investigation; H.-C.K., J.P., J.O., M.K., E.J.P., S.H.B., and Y.S.: methodology; H.-C.K., J.P., J.O., E.J.P., and Y.S.: resources; H.-C.K., E.J.P., and Y.S.: software; Y.S.: supervision; H.-C.K., E.J.P., and Y.S.: validation; H.-C.K., E.J.P., and Y.S.: visualization; H.-C.K., E.J.P., and Y.S.: writing – original draft; H.-C.K., J.P., J.O., M.K., E.J.P., S.H.B., and Y.S.: writing – review and editing.

## Conflicts of interest disclosure

No conflicts of interest are to be declared.

## Research registration unique identifying number (UIN)

1. Name of the registry: Clinicaltrials.gov
2. Unique identifying number or registration ID: NCT04981639.
3. Hyperlink to your specific registration (must be publicly accessible and will be checked): https://clinicaltrials.gov/ct2/show/NCT04981639

## Guarantor

Hyun-Chang Kim, Eun Jung Park, and Young Song are guarantors.

## Data availability statement

The data of this investigation is shared in Mendeley Data.

Kim, Hyun-Chang (2023). Analgesic effects of combined transversus abdominis plane block and intramuscular electrical stimulation in patients undergoing cytoreductive surgery followed by hyperthermic intraperitoneal chemotherapy: a randomized controlled trial. Mendeley Data, V1, doi: 10.17632/yr2nppvhkr.1
